# A Unique Presentation of Jaundice

**DOI:** 10.7759/cureus.35397

**Published:** 2023-02-24

**Authors:** Teresa Del Rio, Lloyd Santiago, Raghav Bansal

**Affiliations:** 1 Internal Medicine, Wyckoff Heights Medical Center, New York City, USA; 2 Gastroenterology, Icahn School of Medicine at Mount Sinai, New York City, USA

**Keywords:** jaundice cholestatic, hepato biliary cancers, extrahepatic biliary cancers, biliary diseases, advanced gastric cancer

## Abstract

Obstructive jaundice has many common etiologies, which might cause us to overlook diagnoses that are far worse. This is a case of a Hispanic male who presented with jaundice and worsening liver function. During his hospitalization, he was erroneously diagnosed before being diagnosed with gastric cancer. Even though biliary obstruction has many common etiologies, there are cases that can’t be explained, which warrants further investigation such as malignant etiologies.

## Introduction

Jaundice can be described as the yellowing of the skin on physical exam. This is particularly caused by elevated bilirubin due to hepatocellular disease, impaired canalicular excretion of bilirubin, or biliary obstruction. Obstructive jaundice can be caused by several etiologies like primary sclerosing cholangitis, hepatic lymphoma, parasitic infections (e.g., Ascariasis), and causes of pancreatitis, which include gallstones and chronic ethyl alcohol (ETOH) consumption [1,2]. Malignancies such as gastric carcinomas that metastasize to the liver are well-known. Linitis plastica (LP) is a gastric carcinoma not well-identified and is rare. It has been described as macroscopically thickened; pathophysiology was described as fibrous tissue, causing the walls of the stomach to become thick, hard, and rubbery. It is described as a carcinoma with scirrhous stroma, involving thickening of the submucosal and muscular layer of the stomach, involvement of mucosal alteration is variable. In current studies, LP is usually poorly differentiated and originates in the gastric gland, and shows extensive submucosal invasion. Furthermore, metastatic LP can also be found, and several routes have been described, including hematogenous metastases, lymphatic spread, and direct extension. Metastatic LP is indistinguishable from that of primary scirrhous carcinoma of the stomach [3-5]. Metastatic LP has been described with lobular breast cancer, non-Hodgkin lymphoma, omentum malignancy, and in rare cases, uterine malignancy [6-8]. 

LP can usually be accompanied by fast peritoneal dissemination, considerable lymph node metastasis, and direct invasion into surrounding organs [3]. For this reason, the disease is hard to diagnose until it has progressed to the level of symptom detection. There are two distinct types of LP as discussed in various studies, “giant-fold or waffle-like” and flat type. Waffle-like mucosa is described as changing to enhance the mucosal folds [3]. This type has been studied and has been found to have a slower progression with a prognostic of three to five years with a later “fast” progression on average seen in one year [3-5]. The second subtype known as flat type most commonly originates from the antrum, near the lesser curvature, and then extends to involve the antrum circumferentially. This type has not been widely studied, possibly to the faster progression of the malignancy [3,9-12].

Carcinomas of the liver are primarily differentiated into primary liver cancers versus metastases. Most of the carcinomas of the liver are metastatic in origin. Primary liver cancers include hepatocellular carcinoma and cholangiocarcinoma versus metastatic. Liver cancers can cause infiltrative disease versus a space-occupying lesion [13-15]. In metastasis, the most common spread of cancer is from colon cancer, gastric cancer, and melanoma [15]. Differentiation of types of liver cancer is primarily done with biopsy, in history, most of the primary liver cancers are associated with chronic viral hepatitis and cirrhosis of different origins [14-15].

## Case presentation

A 36-year-old Hispanic male presented to the Emergency Room twice with abdominal pain, nausea, and vomiting. The physical exam was remarkable for scleral icterus and abdominal tenderness. Laboratory evaluation showed high lipase levels and abnormal liver function test. Initial laboratory showed lipase 4,693 U/L, total bilirubin 12.3 mg/dl, direct bilirubin 9.47 mg/dl, alkaline phosphatase 888 U/L, aspartate transaminase (AST) 482 U/L, and alanine transaminase (ALT) 499 U/L. The patient was admitted with acute pancreatitis, a suspected biliary source.

Endoscopic retrograde cholangiopancreatography (ERCP) was unsuccessful due to severe inflammation of the stomach and ampulla, leading to failed cannulation. Despite medical treatment, the patient was not clinically improving with worsening liver function test, lipase 5070 U/L, alkaline phosphatase 206 U/L, AST 1303 U/L, ALT 247 U/L, and total bilirubin 13.4 mg/dl. CT of the abdomen and pelvis with IV contrast showed irregular thickening of the gastric wall, increased intra- and extrahepatic biliary duct dilation with persistent peri-pancreatic fluid and stranding, and the liver was homogeneous with no focal mass. Subsequently, a hepatobiliary iminodiacetic acid scan (HIDA) reported high-grade biliary obstruction. A decision was made to perform a percutaneous transhepatic cholangiography and percutaneous biliary drainage. The procedure was successful for biliary drain balloon sweep of the distal common bile duct and the biliary drain was left in place, draining bile.

Regardless of this intervention, the patient's symptoms continued to worsen as well as liver enzymes. Subsequently, the patient underwent esophagogastroduodenoscopy (EGD) due to declining hemoglobin levels, persistent nausea, and poor oral intake. EGD showed congested and erythematous friable mucosa on the stomach, which prompted the need for a biopsy of the tissue. Biopsy was consistent with signet-ring cell carcinoma and diagnosed as LP. Repeat CT abdomen and pelvis with IV contrast showed interval apparent progression of diffusely heterogeneous appearance of the hepatic parenchyma, with marked enlargement of a diffuse hypoenhancing infiltrative mass. This strongly raised the possibility of the progression of metastatic disease. The patient was not deemed a candidate for treatment due to the advanced stage of the malignancy gastric cancer with metastasis to the liver. The patient's condition deteriorated, requiring intubation. At this point, a palliative consult was recommended due to the poor prognosis.

## Discussion

Although obstructive jaundice could be explained by numerous conditions, biliary tract disease is one of the most common causes of jaundice. Rare pathologies like malignancy including gastric cancer should be considered on differential diagnosis since they can present with similar symptoms such as abdominal pain and loss of appetite. Initial presentation of previous symptoms alongside elevated lipase levels, transaminitis, and hyperbilirubinemia with direct bilirubin dominance pointed out to pancreatitis of biliary source in our patient.

Elevated lipase levels exceeding three times or more the upper limit of normal (UNL) can be categorized as reduced clearance or physiological causes, intra-abdominal pancreatic and non-pancreatic causes (non-traumatic), critical illness, and malignancy among others [7,14]. Elevated lipase is most commonly seen in pancreatic disease but can also be elevated in pathologies with reduced lipase clearance as in liver cirrhosis and renal impairment conditions [7]. Also, lipase levels can be elevated in biliary tract pathologies such as cholecystitis and cholangitis, and other intra-abdominal conditions without pancreatic causes like peptic ulcer disease, bowel obstruction, or necrosis [3,9-12]. Critically-ill patients can exhibit elevation of lipase levels such as in multi-organ failure, traumatic injuries, or neurosurgical patients with traumatic brain injuries. Drugs such as dipeptidyl peptidase‐4 (DPP‐4) inhibitors and opioids, HIV infection, and conditions such as sarcoidosis and inflammatory bowel disease (IBD) it has been reported that can cause elevated serum lipase as well [1-3].

**Figure 1:**
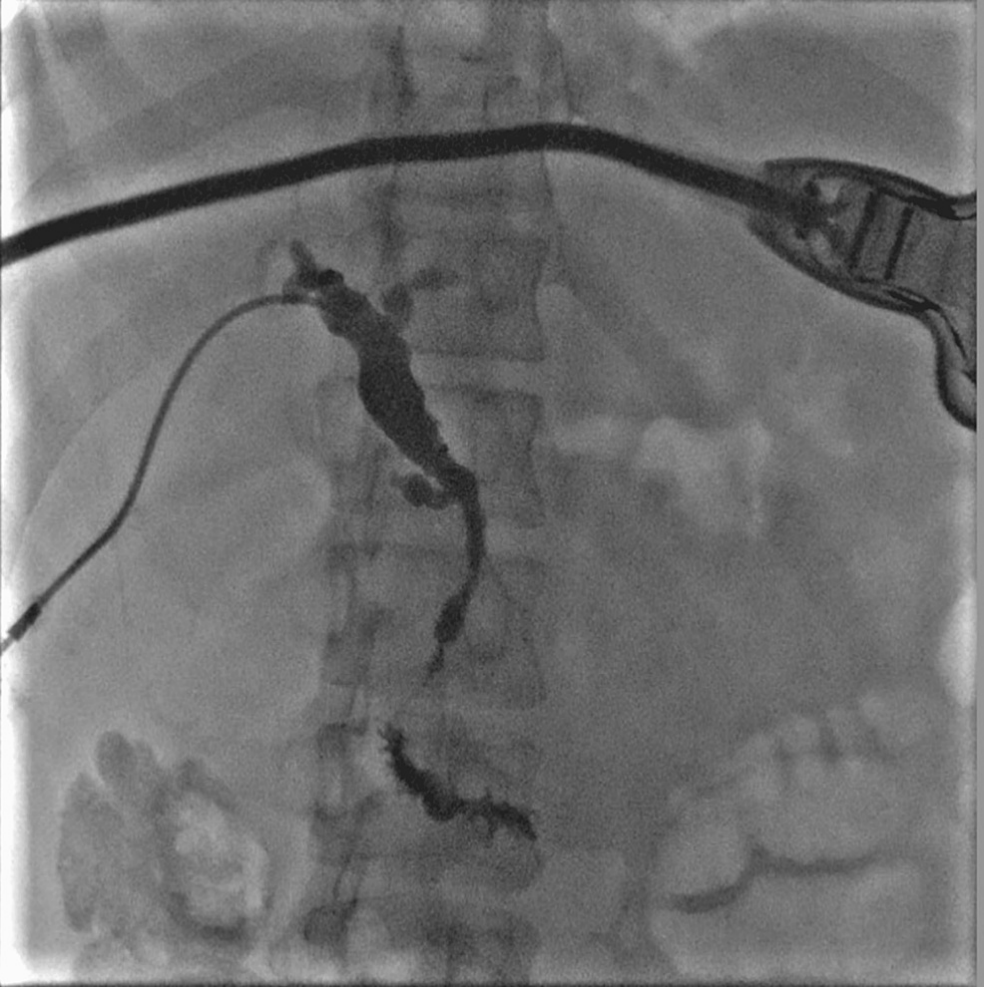
Cholangiogram

Studies show that ERCP has a better success rate and fewer complications than percutaneous transhepatic biliary drainage (PTBD) if biliary obstruction is suspected. In this patient's case, he underwent PTBD with a cholangiogram (Figure 1). However, evidence demonstrates ERCP is associated with a 2-15% risk of pancreatitis [5,10]. The presence of edematous ampulla in this patient distorted the anatomy, making him more susceptible to ERCP-induced pancreatitis. Our patient successfully underwent PTBD.

**Figure 2:**
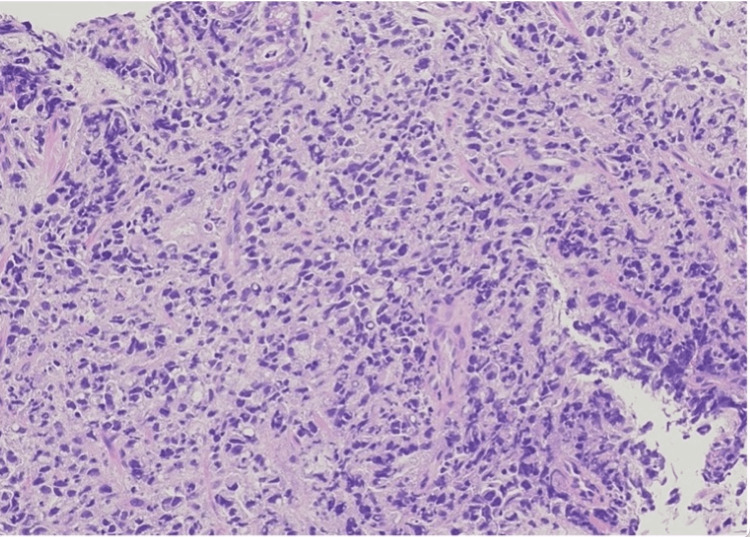
Pathology of gastric antral mucosa infiltrated by diffuse sheets of malignant cells with significant nuclear pleomorphism and signet ring morphology

The culprit of this patient's demise was established to be LP, which as discussed earlier is notorious and can easily be misdiagnosed until biopsy (Figure 2). Persistently elevated liver function tests were caused by metastatic infiltration to the liver. As described, LP metastases fast mostly with peritoneal involvement followed by lymph nodes and peripheral organs including the liver. In this case, it was diagnosed gastric as primary with metastases to the liver. Even prompt diagnosis, in this case, did not have a good prognosis as tumors that fall under the definition of LP have a unique behavior and are believed to be more aggressive than other tumors, with very fast lymph nodes and peritoneal involvement. The prognosis is described as dismal, due to the late presentation of the disease with a median survival rate of 5.7-13.2 months [11,12]. In this case, even a prompt diagnosis of the cause of the elevated bilirubin would not have changed the outcome. 

Incidence and mortality rates of gastric cancer are predominant in Asia, particularly in Japan. According to the International Agency for Research on Cancer (2008), Japan, South Korea, and China contained over 60% of gastric cancer patients, most of whom were 40 years or younger [4]. LP has a median survival of 14 months, which is significantly lower compared to that of gastric adenocarcinoma, which is 62 months [13]. In Latin America, it has been found that populations living in high altitudes, specifically clusters identified in the Andes Mountains, have a higher predominance to develop gastric cancer. It has mostly been associated with low socioeconomic status and decreased access to healthcare [14]. As seen in our case, not all causes of elevated bilirubin are biliary or liver in origin. An occult malignancy should be considered in a patient with an unclear etiology of jaundice. A liver biopsy can be considered to establish the diagnosis. 

## Conclusions

It is important to keep in mind that not all elevated bilirubin levels and lipase levels are secondary to obstruction, as in this unique case it could be secondary to malignancy that has infiltrated the liver. As seen in the present case, LP is a rare malignancy that is known to infiltrate the organs closest to the stomach, thus causing elevated bilirubin and lipase level. Without clear evidence of obstruction causing jaundice, patients should not be put through unnecessary procedures and should be worked up further. As seen in our case, not all causes of elevated bilirubin are biliary or liver in origin. An occult malignancy should be considered in a patient with an unclear etiology of jaundice. 

## References

[1] Reisman Y, Gips CH, Lavelle SM, Wilson JH (1996). Clinical presentation of (subclinical) jaundice--the Euricterus project in The Netherlands. United Dutch Hospitals and Euricterus Project Management Group. Hepatogastroenterology.

[2] Feng Y, Zhang S, Guo T, Zheng W, Wu D, Wu X, Yang A (2020). Validity and safety of corticosteroids alone without biliary stenting for obstructive jaundice in autoimmune pancreatitis. Pancreatology.

[3] Agnes A, Estrella JS, Badgwell B (2017). The significance of a nineteenth century definition in the era of genomics: linitis plastica. World J Surg Oncol.

[4] Katai H, Ishikawa T, Akazawa K (2018). Five-year survival analysis of surgically resected gastric cancer cases in Japan: a retrospective analysis of more than 100,000 patients from the nationwide registry of the Japanese Gastric Cancer Association (2001-2007). Gastric Cancer.

[5] Oguro Y (1994). Endoscopic diagnosis of scirrhous gastric carcinoma (Article in Japanese). Gan To Kagaku Ryoho.

[6] Nakamura K, Kato Y, Misono T (1980). Growing process to carcinoma of linitis plastica type of the stomach from cancer-development. Stomach Intest.

[7] Teplick SK, Flick P, Brandon JC (1991). Transhepatic cholangiography in patients with suspected biliary disease and nondilated intrahepatic bile ducts. Gastrointest Radiol.

[8] Aranha GV, Georgen R (1989). Gastric linitis plastica is not a surgical disease. Surgery.

[9] Matsukawa M, Kurihara M, Hirashima M, Iwasaki Y, Hamada T (1994). Radiological findings of gastric scirrhous cancer (Article in Japanese). Gan To Kagaku Ryoho.

[10] Kohli Y, Takeda S, Kawai K (1981). Earlier diagnosis of gastric infiltrating carcinoma (scirrhous cancer). J Clin Gastroenterol.

[11] Endo K, Sakurai M, Kusumoto E, Uehara H, Yamaguchi S, Tsutsumi N, Ikejiri K (2012). Biological significance of localized Type IV scirrhous gastric cancer. Oncol Lett.

[12] Mastoraki A, Papanikolaou IS, Sakorafas G, Safioleas M (2009). Facing the challenge of managing linitis plastica review of the literature. Hepatogastroenterology.

[13] Sarriugarte-Lasarte A, García-Alberdi E, Pérez-Fernández S (2022). Linitis plastica: current prognostic implication of a classic concept. Cir Cir.

[14] Goodman ZD (2007). Neoplasms of the liver. Mod Pathol.

[15] Montero-Oleas N, Núñez-González S, Simancas-Racines D (2017). The remarkable geographical pattern of gastric cancer mortality in Ecuador. Cancer Epidemiol.

